# NPInter v4.0: an integrated database of ncRNA interactions

**DOI:** 10.1093/nar/gkz969

**Published:** 2019-10-31

**Authors:** Xueyi Teng, Xiaomin Chen, Hua Xue, Yiheng Tang, Peng Zhang, Quan Kang, Yajing Hao, Runsheng Chen, Yi Zhao, Shunmin He

**Affiliations:** 1 Key Laboratory of RNA Biology, Center for Big Data Research in Health, Institute of Biophysics, Chinese Academy of Sciences, Beijing 100101, China; 2 University of Chinese Academy of Sciences, Beijing 100049, China; 3 Guangdong Geneway Decoding Bio-Tech Co. Ltd, Foshan 528316, China; 4 Bioinformatics Research Group, Key Laboratory of Intelligent Information Processing, Advanced Computing Research Center, Institute of Computing Technology, Chinese Academy of Sciences, Beijing 100190, China

## Abstract

Noncoding RNAs (ncRNAs) play crucial regulatory roles in a variety of biological circuits. To document regulatory interactions between ncRNAs and biomolecules, we previously created the NPInter database (http://bigdata.ibp.ac.cn/npinter). Since the last version of NPInter was issued, a rapidly growing number of studies have reported novel interactions and accumulated numerous high-throughput interactome data. We have therefore updated NPInter to its fourth edition in which are integrated 600 000 new experimentally identified ncRNA interactions. ncRNA–DNA interactions derived from ChIRP-seq data and circular RNA interactions have been included in the database. Additionally, disease associations were annotated to the interacting molecules. The database website has also been redesigned with a more user-friendly interface and several additional functional modules. Overall, NPInter v4.0 now provides more comprehensive data and services for researchers working on ncRNAs and their interactions with other biomolecules.

## INTRODUCTION

RNAs are not just intermediate molecules between DNA and protein. Over the recent decades, large numbers of noncoding RNAs (ncRNAs) have been found that do not encode for proteins and instead play regulatory roles by interacting with biomolecules. For example, the lncRNA Xist mediates X chromosome silencing through an interaction with chromatin DNA (1). MicroRNAs interact with the 3′ UTR region of their target mRNAs and regulate their post-transcriptional repression (2). Studying the ncRNA interactions is thus important for understanding the regulatory network among biomolecules.

With the advancement of high-throughput sequencing technology, a number of new methods have been developed to investigate interactions pertaining to RNAs. Approaches such as CLIP-seq (3), PARIS (4), CLASH (5), ChIRP-seq (6) and GRID-seq (7) have the ability to globally find the interacting partners of specific target ncRNAs. We initially established the NPInter database (8) in 2006 in order to organize and classify such ncRNA interactions and have later upgraded and expanded the database for the past 10 or so years (9,10). In the former version NPInter v3.0 (2016), we already added interactions detected by newly developed methodology, and included functional modules such as binding prediction and network viewing to facilitate its usage. However, since the release of NPInter v3.0, quite a number of articles related to ncRNA interactions have been published, accompanied by a large amount of high-throughput sequencing data. This paper thus describes the update of NPInter to the fourth edition, which includes the integration of newly identified ncRNA interactions and even more user-friendly web services. Specially, we first included circular RNA (circRNA) interactions and ncRNA–DNA interactions detected by ChIRP-seq data. Information on diseases associated with each biomolecule and interaction was collected to improve their function annotation. We also redesigned the entire website to provide a more friendly user interface. In order to assist users in coping with the dramatically increased data size, the new browse module now contains several convenient ways for users to search for target interactions. All interaction data can be freely downloaded from the download page.

## DATA COLLECTION AND ANNOTATION

**Figure 1.**
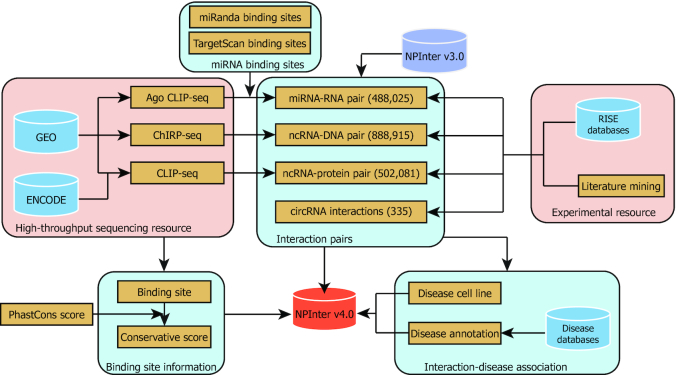
An overview of the NPInter v4.0 data integration.

For the NPInter v4.0, we collected interaction data primarily through manual literature mining and processing of high-throughput sequencing data. Data from different sources were subsequently integrated with redundant entries removed. For the convenience of searching, we annotated the involved biomolecules with commonly used molecule IDs. An overview of data integration workflow is shown in Figure 1.

### Interactions recorded in literature and databases

To search for experimentally validated ncRNA interactions, we conducted mining of literature published between April 2015 and April 2019 and found 1221 papers. The keywords used in searching the PubMed database are listed in the Supplementary Material. We only included interactions with experimental evidence. Besides, interaction data from the RISE database (11) were integrated into NPInter v4.0.

### RNA–protein interactions from CLIP-seq processing

We searched the Gene Expression Omnibus (GEO) (12) for CLIP-seq datasets released in the past 4 years and downloaded raw sequencing data of 69 datasets. We also downloaded 338 ENCODE eCLIP datasets (13). With these CLIP-seq data, we mapped the reads to the reference genome (hg19 for human, mm9 for mouse) using GSNAP (14) and called peaks with the Piranha software (15). We compared the RNA–protein binding sites with ncRNA annotations from the NONCODE v5 database (16) and assigned a NONCODE ID to each site overlapping an ncRNA. The binding score for each ncRNA–protein interaction was then calculated using LncPro (17). We also computed the mean PhastCons sequence conservation score (18) of the binding sites for each ncRNA.

### miRNA target extraction from Argonaute CLIP-seq

For Argonaute CLIP-seq datasets, raw data were processed as described earlier. We then used BEDOPS (19) to identify the TargetScan (20) and miRanda (21) miRNA binding sites that overlapped with Argonaute binding peaks. RNAs with predicted target sites that also located to Argonaute CLIP-seq peaks were believed to interact with the miRNAs for which the targets were predicted.

### RNA–DNA interactions from ChIRP-seq processing

In NPInter v4.0, we have included RNA–DNA interactions derived from all ChIRP-seq datasets published on the GEO database (12). The HISAT2 software (22) was employed to map ChIRP-seq DNA fragments to the reference genome and peak calling was then done by MACS2 (23). Genome binding sites were annotated with GENCODE annotations (e.g. UTR, intron, and exon) (24), while sites in intergenic regions were annotated with the nearest gene. To visualize the genome-wide distribution of the interactions, we counted the number of interactions inside each 1 Mb window across the genome and visualized it by a heatmap using BioCircos.js (25).

### Annotation, redundancy removal and integration

For all biomolecules involved in interactions, we assigned the IDs used by renowned databases. ncRNAs were annotated with NONCODE IDs (16), miRBase IDs (26) or circBase IDs (27), while proteins were annotated with UniProt IDs (28). Ensembl IDs (29), UniGene IDs (30) and RefSeq IDs (31) were also provided where available. Since the data were derived from different sources, we standardized the names of tissues (or cell lines) and experiments. Then, we eliminated redundancies and integrated the repetitive interactions. We combined experiments, tissues, references, sources and binding sites of redundant entries and allocated new interaction IDs for these interactions.

### Disease annotation

Disease annotations were collected from several databases, including LncRNADisease (32), MNDR (33), eDGAR (34) and circRNADisease (35). Biomolecules and their interactions were labeled with the relevant disease information. For RNA–DNA interactions, we also downloaded risk GWAS sites from the GWAS Catalog database (36) and marked the corresponding genome regions with this information.

## DATABASE CONTENT AND SERVICES

### Interactions and associated information

**Figure 2.**
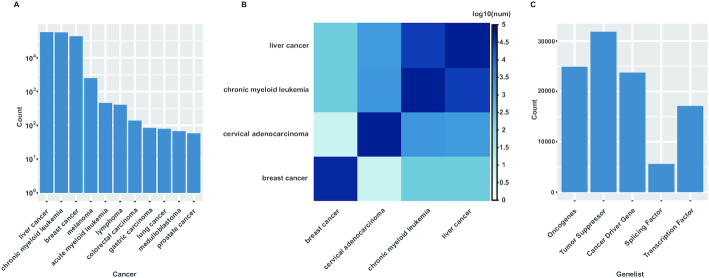
Function analysis using NPInter v4 interactions. (**A**) Counts of cancer cell line-specific interactions. (**B**) Heatmap for overlapped interactions among several types of cancers. Interaction number has been calculated by log 10. The values on the diagonal are the numbers of cancer-specific interactions. (**C**) Counts of interactions searched by gene sets with specific functions.

In the NPInter v4.0, we added to the preexisting 491 416 entries in NPInter v3.0, a total of 609 242 new interactions (not including 888 915 ncRNA–genome binding interactions) obtained from different data sources (see Table 1) and including 35 organisms. These interactions cover most kinds of ncRNAs, including lncRNA, miRNA, circRNA, snoRNA, snRNA, etc. (see Table 2). Using these data, we tried to apply some function analysis. By allocating the interactions to cell types, we found many interactions occurring in over 200 cancer cell lines belonging to 50 kinds of cancers. For some of common cancers, more than 100 tissue-specific interactions were found (Figure 2A). We also found numbers of common interactions across different cancer types (Figure 2B). By using our new Function search module to search interactions with multiple gene lists with certain functions, we also acquired numerous putative interactions related to these functions (Figure 2C).

**Table 1.** Statistics of interactions from different data sources

Data sources	Interactions
Literature mining	9595
High-throughput data (exclude Ago CLIP-seq and ChIRP-seq)	498 083
Predicted miRNA binding with Ago CLIP-seq data	464 043
ChIRP-seq data	888 915
Other database	129 585

**Table 2.** Statistics of different types of ncRNA interactions

Interaction type	Interactions
lncRNA interactions	658 171
miRNA interactions	488 025
snoRNA interactions	61 700
snRNA interactions	12 789
circRNA interactions	335

We furnished each interaction entry with annotations of both molecules as well as detailed annotations of the interactions, including the interaction level, the interaction class, tags, organism, tissue or cell type, experimental description, the interaction description, the data source and binding sites. The interaction level is defined according to the molecular types of the interacting molecules (such as ‘RNA–RNA’, ‘RNA–protein’, etc.). For each binding site, we calculated the average PhastCons (18) score across the nucleotides to represent sequence conservation in different organisms. To access the binding potential of ncRNA–protein interactions captured by the CLIP-seq datasets, we applied the LncPro software (17) to calculate a structure-based binding score. On the Interaction Profile page, users can view all the details described earlier as well as the reference information documenting the interactions.

For each participating molecule, we organized its IDs, molecular type, organism, description, aliases and related diseases into the Molecular Profile page. Users can search for individual molecules and view their details and their interaction partners on this page.

In addition, we integrated some new data sources and annotations, which greatly expanded the coverage of our database. We will describe them in detail in the following paragraphs.

#### ncRNA–DNA interactions

Previous studies have reported that ncRNAs have the ability to interact with genomic DNA and affect transcription. The best known example is the lncRNA Xist, which functions in dosage compensation by interacting with the X chromosome (1). With the accumulation of newly released ChIRP-seq datasets, we included 888 915 RNA–DNA interactions in NPInter v4.0. For each ChIRP targeted ncRNA, we organized the corresponding genome binding sites into a table on the Molecular Profile page.

#### circRNA-associated interactions

Recent evidence suggests that circRNAs play critical roles in diverse biological processes via interactions with other biomolecules (37). The human circRNA CDR1as has 74 miR-7 seed matches that allow it to function as an miR-7 sponge (38). Exon–intron circRNAs can interact with snRNAs and enhance the expression of their parental genes (39). To collect such interactions, we searched the literature for circRNA-associated interactions and integrated them into NPInter v4.0.

#### Disease annotation

Previous research has validated that ncRNAs are associated with a large number of diseases (40,41). In most cases, ncRNAs exert regulatory roles through interactions with molecules involved in diseases. For example, BACE1-AS can form a duplex with BACE1 mRNA and upregulate BACE1 translation in Alzheimer’s disease-related cells (42). These interactions highlight the importance of adding known ncRNA–disease associations to our interaction data. To facilitate ncRNA–disease research, we have therefore included disease annotation for molecules involved in NPInter interactions. Users can find them on both the Interaction Profile page and the Molecular Profile page. Besides, we have provided users with an application to search for associated molecules and interactions using disease names.

### Service update

We redesigned the entire web interface for the NPInter v4.0. The new NPInter UI, which was constructed using Django and Bootstrap, is even more user-friendly and convenient. We also optimized original NPInter modules such as BLAST and Cytoscape. The BLAST module now provides a detailed Results page with links to related ncRNA molecules. Besides, we have substantially updated the Browse module and have added a Biocircos.js module.

#### New browse module

Based on users’ feedbacks, we have learned that many researchers want to search interactions by tissue, interaction class, interaction level and data source. The old NPInter browse module is not convenient for searches on such interactions. In NPInter v4.0, we paid much attention to improving the Browse module. Users can now filter the interactions using multiple parameters such as tissues, organisms, data sources, interaction classes or interaction levels. They can also select multiple types in one field and search for keywords in the filtered results. We thus believe that the new module will allow users to browse the database in a convenient and time-saving manner.

#### Biocircos.js module

In the NPInter v4.0, we have added a large amount of ncRNA–DNA interactions derived from high-throughput data. For each ChIRP targeted ncRNA, we can call thousands of peaks across the whole genome. This makes it difficult for users to obtain an overview of all interactions. To achieve such an overview, we applied our previously developed module Biocircos.js (25) to plot interactions across the entire genome. We linked all hotspots interacting with the molecule and depicted the interaction density among the genome.

#### Function search module

To help user find interactions that function in diseases or important biological processes, we collected some gene lists with certain functions, including oncogenes from ONGene (43), tumor suppressors from TSGene (44), cancer driver genes from 20/20 rule (45) and CGC (46), transcription factors from HOCOMOCO (47) and JASPAR (48), and alternative splicing factors from MiasDB (49). We also processed GDC TCGA cancer expression profiles (50). We filtered differentially expressed lncRNAs with a standard of fold change ≥2 and rank sum test *P*-value ≤0.01. Besides, tissue expression profiles were downloaded from GTEx project (51). We divided the tissue Transcripts Per Million (TPM) by the average TPM of other tissues to calculate a tissue-specific fold change (TSFC). We selected lncRNAs whose TSFC ≥ 8 and TPM ≥ 1 as tissue-specific expressed lncRNAs. We provided searching service with these gene/lncRNA lists in the Function page. We believe the Function search module will facilitate the identification of valuable interactions in various biological processes.

## CONCLUSION

Overall, NPInter v4.0 has significantly increased the data size obtained by adding all recently identified ncRNA interactions reported in the literature and submitted to data collections. We have organized the interaction entries along with detailed annotations and prediction scores. Each associated molecule has been annotated with relevant types of IDs and can be searched with nucleotide sequences by the BLAST module. We have further integrated circRNA interactions and ncRNA–DNA interactions captured by ChIRP-seq data. Numerous ncRNA binding regions on the genome presented by newly added Biocircos.js module extended the coverage of ncRNA regulatory network in NPInter. To highlight the links between ncRNA interactions and diseases, we annotated disease association for participating molecules. The new website interface also provides much more convenient services. Compared to other similar databases such as starBase (52) and RAID (53), we have been more focused on providing detailed annotations for interactions, not just for molecules. Visualization modules and predictive scores are also integrated in order to add confidence to the interactions.

In recent years, research on ncRNAs has been a hotspot in the scientific community. Newly invented high-throughput methods will keep on providing large numbers of interactions from various organisms and cell types. We will regularly update and maintain the database. Together with our online ncRNA research platform, which contains NONCODE (16), CNCI (54) and ncFANs (55), we hope to provide a comprehensive and informative data source on ncRNA interaction network and a series of web services for RNA research spanning from identification to function.

## Supplementary Material

gkz969_Supplemental_File
