# Supplementary material for: NPInter v4.0: an integrated database of ncRNA interactions
Source: Nucleic Acids Res. 2019 Oct 31;48(D1):D160–5. doi: 10.1093/nar/gkz969 (PMC7145607; doi:10.1093/nar/gkz969)
Supplement: gkz969_Supplemental_File [file gkz969_supplemental_file.docx]

**Supplementary Material**

Key words for searching:

ChIRP RNA, ChIRP Seq, circular RNA bind protein, CLIP bind, CLIP interaction, CLIP non coding RNA, CLIP RNA protein, CLIP Seq, CRAC bind, CRAC cross linking, CRAC RNA protein, HITS CLIP, iCLIP, lincRNA bind, lincRNA bind protein, lincRNA protein interaction, lncRNA bind, lncRNA bind protein, lncRNA protein interaction, ncRNA bind, ncRNA bind protein, ncRNA protein interaction, non coding RNA protein interaction, non coding RNA bind protein post transcriptional, noncoding RNA direct bind protein, PAR CLIP, RIP lincRNA, RIP lncRNA, RIP non coding RNA, RIP noncoding RNA, RIP Seq, RNA protein cross linking, RNA-RNA interaction, ChIRP, ChIRP MS, RIP chip, SPLASH, LIGR seq, MARIO, CLASH, hiCLIP, RAP-RNA, RIA seq, CHART bind, CHART interaction, eCLIP, irCLIP, RNA-DNA interaction, RNA-RBP interaction, RNA bind genome, RNA genome interaction, PARIS interaction, PARIS bind, RNA electrophoretic mobility shift assay, RNA pulldown assay, oligonucleotide-targeted RNase H protection assay, FISH co-localization, DNA-RNA FISH
